# *In Vitro* Exposure to *Escherichia coli* Decreases Ion Conductance in the Jejunal Epithelium of Broiler Chickens

**DOI:** 10.1371/journal.pone.0092156

**Published:** 2014-03-17

**Authors:** Wageha A. Awad, Claudia Hess, Basel Khayal, Jörg R. Aschenbach, Michael Hess

**Affiliations:** 1 Department for Farm Animals and Veterinary Public Health, Clinic for Poultry and Fish Medicine, University of Veterinary Medicine, Vienna, Austria; 2 Institute of Veterinary Physiology, Faculty of Veterinary Medicine, Free University of Berlin, Berlin, Germany; Charité, Campus Benjamin Franklin, Germany

## Abstract

*Escherichia coli* (*E. coli*) infections are very widespread in poultry. However, little is known about the interaction between the intestinal epithelium and *E. coli* in chickens. Therefore, the effects of avian non-pathogenic and avian pathogenic *Escherichia coli* (APEC) on the intestinal function of broiler chickens were investigated by measuring the electrogenic ion transport across the isolated jejunal mucosa. In addition, the intestinal epithelial responses to cholera toxin, histamine and carbamoylcholine (carbachol) were evaluated following an *E. coli* exposure. Jejunal tissues from 5-week-old broilers were exposed to 6×10^8^ CFU/mL of either avian non-pathogenic *E. coli* IMT11322 (Ont:H16) or avian pathogenic *E. coli* IMT4529 (O24:H4) in Ussing chambers and electrophysiological variables were monitored for 1 h. After incubation with *E. coli* for 1 h, either cholera toxin (1 mg/L), histamine (100 μM) or carbachol (100 μM) were added to the incubation medium. Both strains of avian *E. coli* (non-pathogenic and pathogenic) reduced epithelial ion conductance (*G*_t_) and short-circuit current (*I*_sc_). The decrease in ion conductance after exposure to avian pathogenic *E. coli* was, at least, partly reversed by the histamine or carbachol treatment. Serosal histamine application produced no significant changes in the *I*_sc_ in any tissues. Only the uninfected control tissues responded significantly to carbachol with an increase of *I*_sc_, while the response to carbachol was blunted to non-significant values in infected tissues. Together, these data may explain why chickens rarely respond to intestinal infections with overt secretory diarrhea. Instead, the immediate response to intestinal *E. coli* infections appears to be a tightening of the epithelial barrier.

## Introduction

*E. coli* infections in chickens and turkeys are an important economic threat to the poultry industry worldwide. Although research has increasingly focused on the pathogenesis of avian pathogenic *Escherichia coli* (APEC) infections, little is known about the reservoirs of these bacteria. Avian pathogenic *E. coli* (APEC) are present in the normal microflora of the intestinal tract and other mucosal surfaces of domestic poultry and wild birds [1]. In addition, pathogenic serotypes, together with non-pathogenic serotypes, can be isolated from the bird's environment and can be transmitted to humans [2]. Avian pathogenic *E. coli* are mostly associated with extraintestinal disease, principally respiratory or systemic infections [3], [4]. A limited number of serotypes, principally O1, O2, O78, O8, and O35 are commonly implicated in avian colibacillosis [5]–[10]. Colibacillosis in mammals is an enteric disease whereas in poultry it causes localized or systemic disease occurring mostly when the host defense is impaired. The acute form of the disease is characterized by septicaemia, resulting in death, while the subacute form coincides with pericarditis, airsacculitis and perihepatitis. Infections of the reproductive tract lead to salpingitis and/or peritonitis with high mortality [11].

The main route for entry of *E.coli* is the respiratory tract following inhalation of dust contaminated with feces [12], [13]. However, the intestine is the most important reservoir of avian pathogenic *E. coli* [14]. The infection strategy of *E. coli* is to colonize a mucosal site, evade host defenses and multiply. Poultry meat contaminated with *E. coli* may then serves as a possible reservoir for human infections.

It was shown in a previous study that an Enteropathogenic *E. coli* (EPEC) isolated from clinically healthy chickens [11] might affect the intestinal barrier function in humans [15]. In humans, EPEC induced a loss of microvillar absorptive surface area [16], [17], and increased epithelial permeability [18]–[20]. *E. coli* also altered aquaporin water channel localization and inhibited sodium, chloride or glucose absorption in human cell culture [16], . Secretory pathways were also affected by an enteropathogenic *E. coli* infection, as alterations in ion transport have been observed at very early time points in Caco-2 cell monolayers [24]. Furthermore, *E. coli* lipopolysaccharide (LPS) challenge of pigs for 48 h impaired nutrient absorption, increased permeability in the intestine, decreased tight junction integrity, increased paracellular movements of molecules and consequently increased susceptibility to additional infections [25]. The maintenance of a healthy gut, in turn, is considered crucial for optimal nutrient absorption and efficient protection against pathogens. Proceeding from the many known effects of EPEC in mammalian intestine and the frequent occurrence of potentially pathogenic *E. coli* strains in chickens, it was hypothesized that the absence of clinical abnormalities in the chicken's intestine carrying *E. coli* does not necessarily exclude alterations in the function of intestinal epithelia.

So far, no experiments have been reported investigating the effects of avian *E. coli* on the ion transport in the chicken gut. In this context the intestinal epithelial response to the secretagogues that drive ion secretion receive special importance in order to characterize the functional impact of *E. coli* on the intestinal epithelial lining. Therefore, the aims of this study were to characterize how an exposure of avian pathogenic and non-pathogenic *E. coli* affects ion transport in the intestinal epithelium of chickens. In addition, the effect of avian *E. coli* on the intestinal epithelial response to cholera toxin and the endogenous secretagogues histamine and carbamoylcholine ( =  carbachol, an acetylcholine receptor agonist) were also investigated.

## Materials and Methods

### Ethics statement

The animal experiments were discussed and approved by the institutional ethics committee of the University of Veterinary Medicine under the license number GZ 68.205/0227-II/3b/2011. All husbandry practices and euthanasia were performed with full consideration of animal welfare.

### Birds and housing

Broiler chickens (male and female), 5 wk of age, weighing 2.0 to 2.5 kg (n = 10) were used in the present study. The broilers were purchased from a local commercial hatchery (Ross-308, Geflügelhof Schulz, Graz, Austria). The birds were housed on wood shavings and were provided with their diets and water, *ad libitum*, for the duration of the experiment. The broilers were fed diets based on wheat, maize, barley, soybean meal, soybean oil, sunflower oil, and a premix with vitamins, minerals, amino acids, salt, and mono-calcium phosphate. The diet contained 22% crude protein (CP), 8.5% fat, 3.3% crude fiber, and 1.4% lysine.

### Ussing chamber setup

The preparation of epithelia and mounting in Ussing chambers were done as previously described [26]. Following injection with a single dose of thiopental (20 mg/kg) into the wing vein of birds, the jugular vein was cut and birds were bled. Immediately after killing, the mid-jejunum was extracted from the birds and opened longitudinally along the mesenteric fixation, washed from intestinal contents with ice-cold buffer solution (see below) oxygenated with carbogen (95% O_2_/5% CO_2_), and the tunica serosa was carefully removed. The epithelial sheets were mounted in Ussing chambers. Epithelial sheets had an exposed area of 1.1 cm^2^ and were incubated with 12 mL of buffer solution on their mucosal and serosal sides. Up to 12 chambers were used for each bird. The present study focused on the mid-jejunal region of the small intestine. The avian jejunum has been described to have more total surface area and is consequently a primary site of nutrient absorption as compared to other regions of the small intestine [27], [28].

The tissues were first incubated in Ussing chambers under open-circuit conditions for 10 min for equilibration and were then short-circuited by clamping them to 0 mV. Under the short-circuit condition, the tissues were exposed *in vitro* to any of the three treatments, that is, control, pathogenic and non-pathogenic *E. coli* at apical (mucosal) side. The tissues were then monitored to note any changes in electrophysiological variables induced by the exposure to *E. coli*. All treatments were given simultaneously on mid-jejunal segment from the same bird. This procedure was repeated with the mid-jejunum from ten different birds.

### Buffer Solutions

The buffer solution used for washing, transport, and incubation of epithelia contained the following chemicals (Sigma-Aldrich Chemie GmbH, in mmol/L): NaCl, 115; KCl, 5; CaCl_2_, 1.5; MgCl_2_, 1.2; NaH_2_PO_4_, 0.6; Na_2_HPO_4_, 2.4; L-glutamine, 1; Na-D/L-lactate, 5; HEPES-free acid, 10; NaHCO_3_, 25; and mannitol, 10 (320±5 mosmol/kg; pH 7.4). The serosal bathing solution contained 10 mM glucose and was balanced osmotically on the mucosal side with 10 mM mannitol. The incubation medium was continuously gassed with carbogen, and the temperature of the mixture was kept at 38°C by thermostated water jackets. Continuous oxygenation provided recirculation of the incubation solutions with carbogen by means of a gas lift.

### Electrophysiological Measurements

Short-circuit currents (I_sc_) and transepithelial tissue conductances (G_t_) were measured using a computer controlled voltage-clamp device (Muβler Ingenieurbüro für Mess-und Datentechnik, Aachen, Germany). Tissue conductance (*G*_t_ in mS/cm^2^) was determined by measuring the changes in transepithelial potential difference upon short bipolar current impulses (*G*_t_ = Δ*I*/Δ*PD*).

The basal measurements of *I*_sc_ and *G*_t_ were taken after a stabilization period of 20 min (low/or no fluctuation of the measurements). Non-pathogenic *Escherichia coli* IMT11322 (Ont:H16) was isolated from the intestine of a clinically healthy chicken and was used as a non-virulent negative control. The isolate belongs to phylogenetic group A and sequence type 10. This non-pathogenic *E. coli* harbours only adhesion-related genes F1-fimbriae (fimC) and curli fimbriae (csgA) and did not harbour any of the virulence genes. Avian pathogenic *E. coli* IMT4529 (O24: H4), was obtained from the internal organs of a laying hen clinically diagnosed with systemic APEC infection (colibacillosis), belonging to phylogenetic group ABD and sequence type 117. This pathogenic strain possesses a broad range of virulence-associated genes typical of animal- and human-derived extraintestinal pathogenic *E. coli* strains, including adhesion genes, iron-acquisition genes and serum resistance and protectin genes, invasion related genes and toxin gene (vacuolating toxin). Furthermore, this pathogenic strain was demonstrated to induce Colibacillosis in chickens [29]. Micro-organisms were routinely grown in Lennox L Broth Base (Invitrogen, California, USA) (LB)-broth at 37°C for 24 h in a shaking incubator. *Escherichia coli* CFU count was determined from the suspension by serial dilutions in duplicate using LB agar. For use in Ussing chamber, *E. coli* suspensions from the same stock solution were centrifuged for 5 min at 10000 rpm. The pellets were washed 3 times by Ussing buffer solution. Afterwards the pellet was resuspended and centrifuged again at the same conditions mentioned above. Finally, the pellets were resuspended in Ussing buffer and used in the Ussing chamber at the dose 6×10^8^ CFU/mL as previously described [3], [30].

When the tissue had stabilized, i.e. after recording the basal *I*_sc_ and *G*_t_, *E. coli* (6×10^8^) was added to the mucosal side, and *I*_sc_ and *G*_t_ were monitored for 1 h. Simultaneously, control tissues were incubated without *E. coli* to obtain data for time-dependent changes in *I*_sc_ and *G*_t_. The effects of *E. coli* application to the mucosal side on the electrical variables are given as the changes in *G*_t_ or *I*_sc_ (Δ*G*_t_ or Δ*I*_sc_), which were calculated for each tissue as the difference between the *G*_t_ or *I*_sc_ at a given time after challenge with *E. coli* and the basal steady state value of *G*_t_ or *I*_sc_. Thereafter, cholera toxin (10 mg/L), which acts similar to the heat-labile enterotoxin of *E. coli*, was added to the mucosal side after pre-incubation of the tissues with or without *E. coli* for 1 h. At the end of the experiment, 100 μl of buffer solution containing *E. coli* was inoculated onto LB agar for confirming the viability and presence of *E. coli.*

Furthermore, histamine and carbamoylcholine (100 μmol/L), as neural and/or immune mediators that commonly elicit chloride secretion, were tested for their influence on *I*_sc_ and *G*_t_ after infection. Carbachol as an acetylcholine analogue stimulates intracellular calcium release, thereby activating potassium channels, calcium-dependent chloride channels and, via Cystic Fibrosis Transmembrane Conductance Regulator (CFTR) channels. Histamine or carbamoylcholine were applied basolaterally after 1 h of incubation with *E. coli* (or the corresponding time point in control tissues) and the tissues were further incubated for at least 30 min. In the present study, we focused on histamine and carbachol, because, it is known that receptors mediating the action of those agonists are widely distributed in the gastrointestinal tract [31].

### Statistical Analysis

All data were included in a descriptive analysis. Normal distribution was confirmed by Kolmogorov-Smirnov's test. Data are presented as means with standard error of means (SEM). Statistical significance was designated as *P*≤0.05. Multiple groups were compared using the one-way analysis of variance (one-way ANOVA) and statistically different means (*P*<0.05) were further separated using Least Significant Difference (LSD) and Duncan's Multiple Range Test. The I_sc_ and G_t_ values at individual time points of each bird were averaged to obtain animal means and these were taken to calculate group means. Time-dependent changes within groups were assessed by repeated-measures ANOVA and subsequent paired *t*-test to evaluate the effect of *E. coli* application on *I*_sc_ and *G*_t_. All tests were performed using appropriate software (PASW statistics 20, SPSS, Chicago, IL, USA).

## Results

### Responses to *E. coli* Application

The basal values of G_t_ and I_sc_ were not significantly different between the treated epithelia. However, there were some relative changes in values, which may be due to differences in expression or regulation of sodium and chloride channels along the mid-jejunum, because the electrical current across the epithelium results mainly from the absorption of Na^+^ and secretion of Cl^−^.

**Figure 1:**
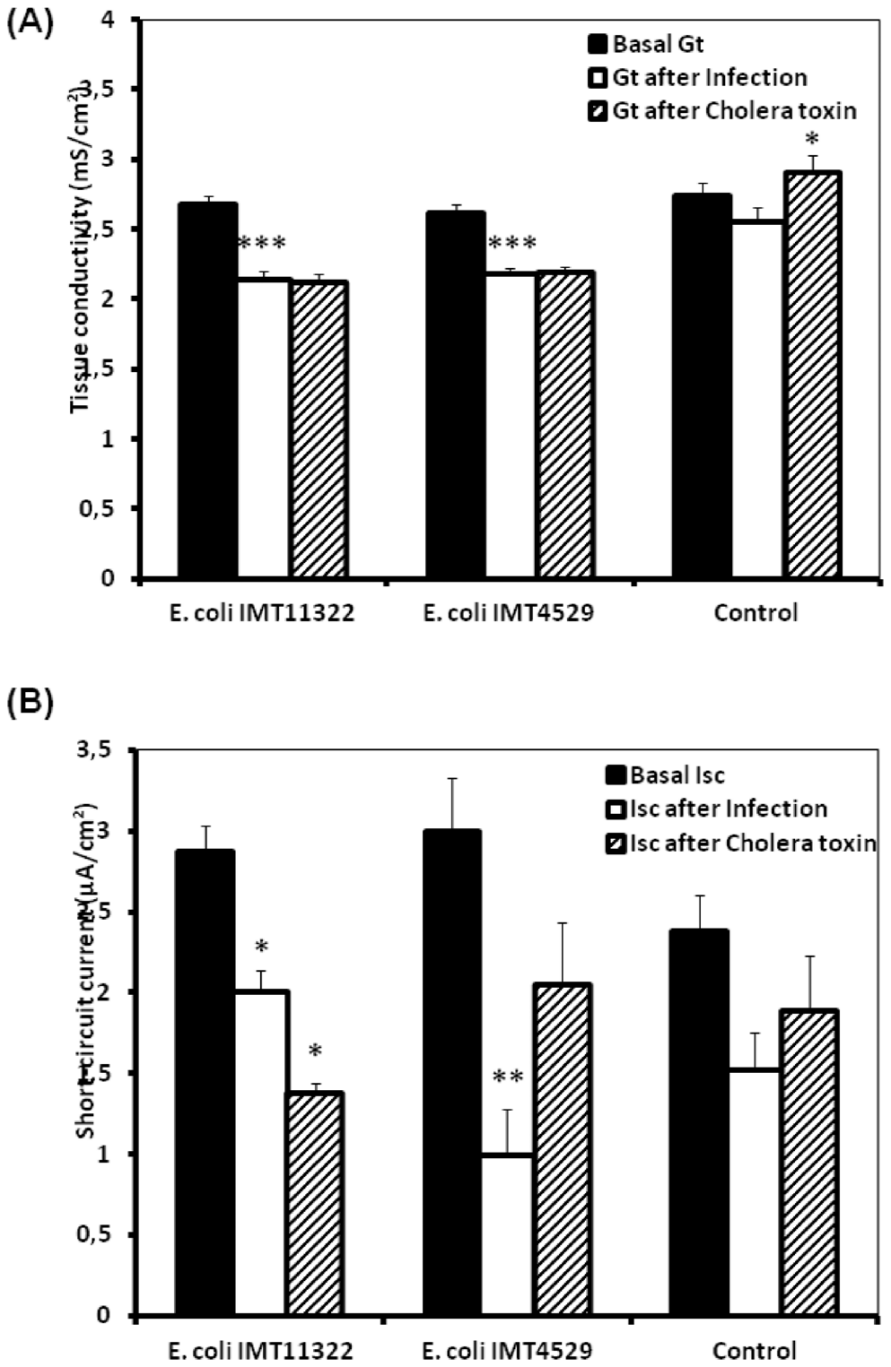
Effect of luminal avain *E. coli* on the permeability (*G*_t_) and short-circuit current (*I*_sc_) of isolated jejunal epithelial sheets from 6-wk-old broiler chickens by the Ussing chambers technique. The electrophysiological changes of jejunal tissues exposed to *E. coli* and cholera toxin at the luminal side were monitored. (A) Permeability (*G*_t_), (B) Short-circuit current (*I*_sc_), black columns represent basal values before additions while white columns represent values 1 h after addition of *E. coli (*avian non-pathogenic, IMT11322 and avian pathogenic, IMT4529) and oblique lines columns represent values 30 min after addition of cholera toxin. Data from simultaneously incubated epithelia without infection served as controls. Data are given as means + SEM (n =  10). *^/^**/***Asterisks mark significant differences (*P*<0.05/*P*<0.01/*P*<0.001).

**Figure 2:**
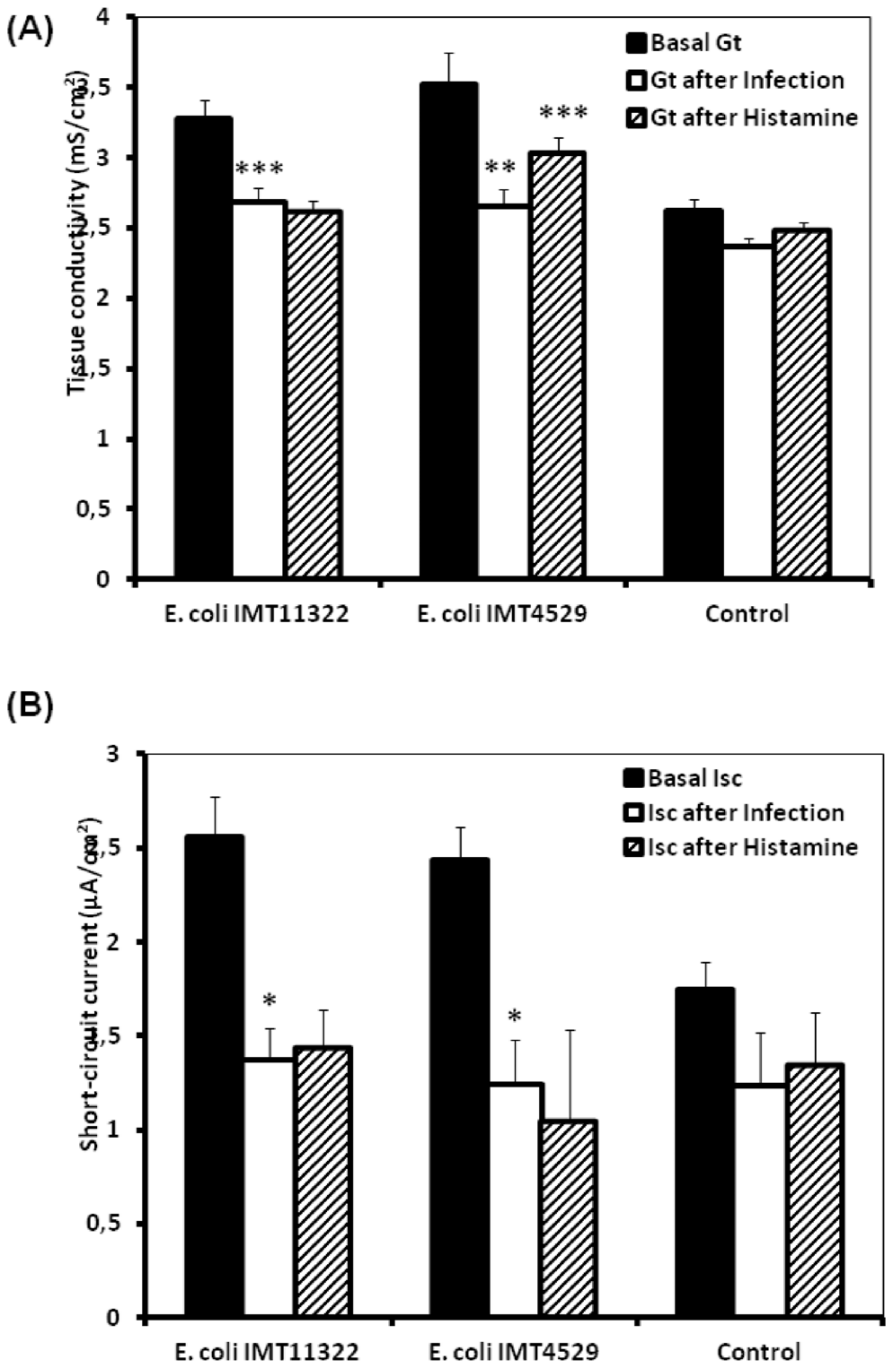
The changes of permeability (*G*_t_) and short-circuit current (*I*_sc_) of jejunal epithelial sheets of broiler chickens after exposure to avain *E. coli* on the luminal side and histamine on the serosal side. (A) Permeability (*G*_t_), (B) Short-circuit current (*I*_sc_), black columns represent basal values before additions while white columns represent values 1 h after addition of *E. coli* (avian non-pathogenic, IMT11322 and avian pathogenic, IMT4529) and oblique lines columns represent values 30 min after addition of histamine. Data from simultaneously incubated epithelia without infection served as controls. Data are given as means + SEM (n =  10). *^/^**/***Asterisks mark significant differences (*P*<0.05/*P*<0.01/*P*<0.001).

**Figure 3:**
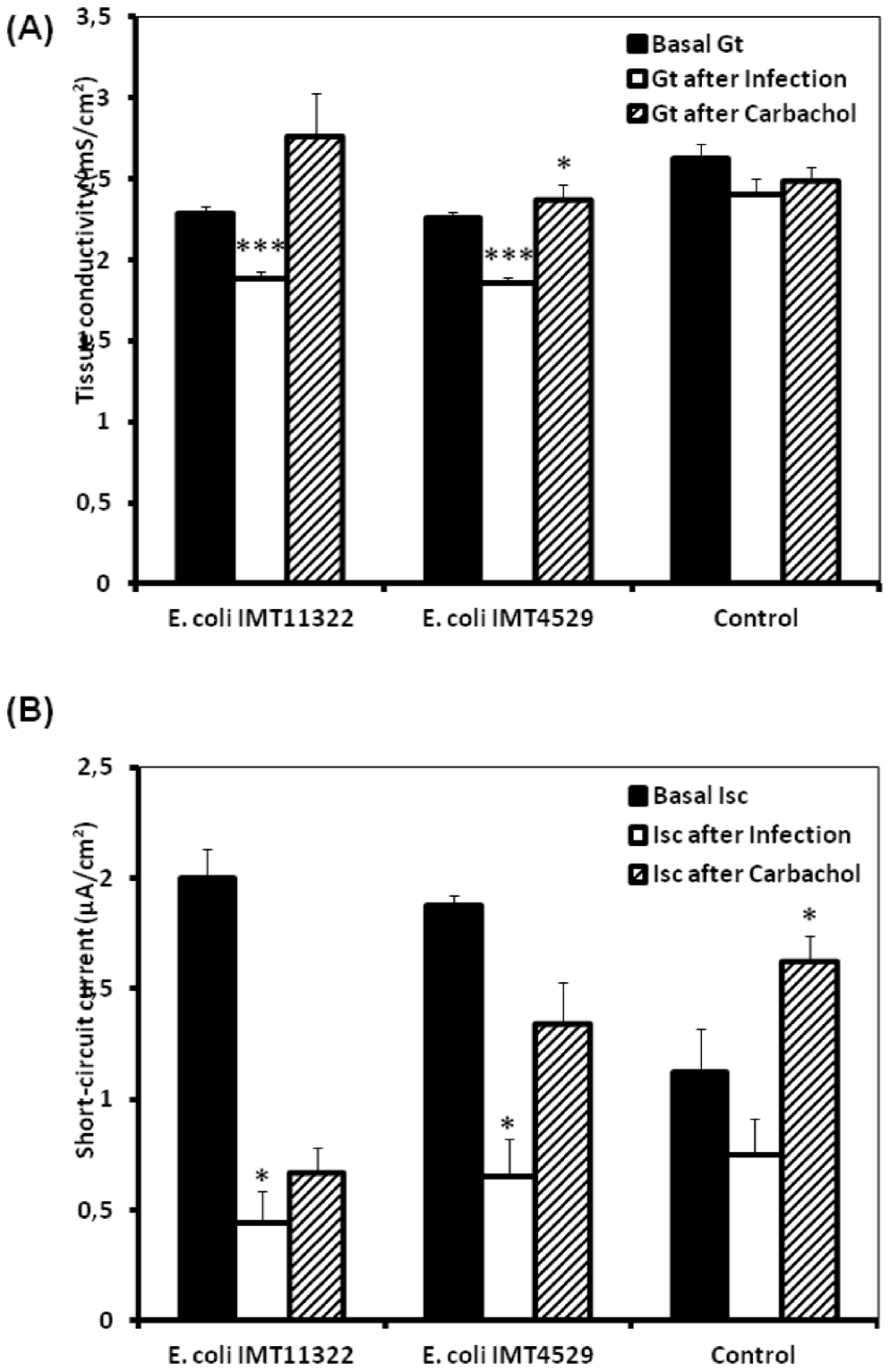
The changes of permeability (*G*_t_) and short-circuit current (*I*_sc_) of jejunal epithelial sheets of broiler chickens after exposure to avain *E. coli* on the luminal side and carbachol on the serosal side. (A) Permeability (*G*_t_), (B) Short-circuit current (*I*_sc_), black columns represent basal values before additions while white columns represent values 1 h after addition of *E. coli* (avian non-pathogenic, IMT11322 and avian pathogenic, IMT4529) and oblique lines columns represent values 30 min after addition of carbamoylcholine (carbachol). Data from simultaneously incubated epithelia without infection served as controls. Data are given as means + SEM (n =  10). *^/^**/***Asterisks mark significant differences (*P*<0.05/*P*<0.01/*P*<0.001).

**Figure 4:**
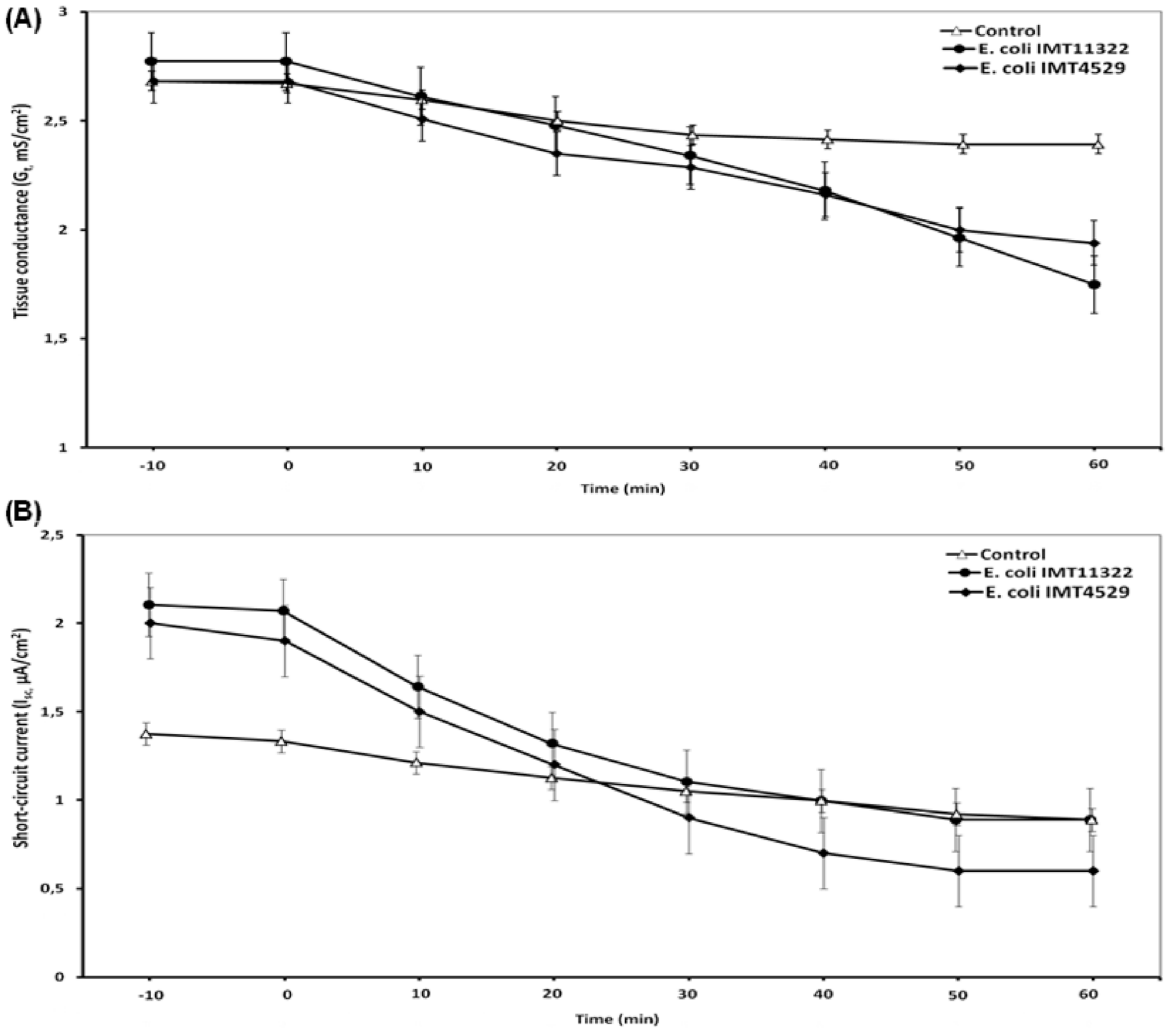
Time course of the effects of the mucosal exposure to living *E. coli* (avian non-pathogenic, IMT11322 and avian pathogenic, IMT4529) on the tissue ionic conductance (G_t_) and short-circuit current (I_sc_) of jejunal epithelial sheets of broiler chickens mounted in Ussing chambers. (A) Permeability (*G*_t_), (B) Short-circuit current (*I*_sc_), results are expressed as means ± SEM [n =  30 (number of experiments for each treatment)].

Generally, in Ussing chambers, the buffer allowed the survival of both bacterial strains until the end of the experiment. The exposure to a pathogenic *E. coli* strain to the jejunum decreased the *G*_t_ (*P*<0.01; Table 1). A comparable drop in *G*_t_ was also induced by the non-pathogenic *E. coli*. The latter implies that the decrease in *G*_t_ was a general response to *E. coli* rather than a specific pathogen-induced response. The decrease in *G*_t_ coincided with a decrease in net charge transfer across the epithelium, evidenced by decreased *I*_sc_ after both *E. coli* applications (*P*<0.05; Figure 1, 2, 3, 4). Table 1 lists the absolute and relative changes in *G*_t_ and *I*_sc_ from baseline values in comparison to untreated control tissues. The absolute and relative changes in *G*_t_ and *I*_sc_ were not different between the pathogenic vs. non-pathogenic *E. coli*-treated epithelia. However, both *E. coli*-treated tissues had significantly larger decreases in *G*_t_ and *I*_sc_ compared to the untreated control tissues, indicating that the transmural ion conductance, a measure of epithelial barrier function, was reduced in the infected jejunal tissues. The results indicated that both pathogenic and non-pathogenic *E. coli* altered the active transepithelial ion transport and ionic permeability in a way that the intestinal epithelium became tighter to ion permeation following infection.

**Table 1:** Basal values of transmural conductivity (*G*_t_) and short-circuit current (*I*_sc_) of isolated jejunal mucosa of broiler chickens and their changes in response to *Escherichia coli* application.

	Groups					
Electrophysiological variable^1^	*E. coli* IMT11322	E. *coli* IMT4529	Control	SEM^2^		*P*-value
**Tissue conductivity (** ***G*** **_t_)**						
** Basal ** ***G*** **_t_ (mS/cm** 2 **)**	2.75	2.68	2.67	0.09		0.936
***E. coli*** ** 1 or ** ***E. coli*** ** 2 application** 3						
**Δ** ***G*** **_t_ after 1 h (mS/cm** 2 **)**	−0.51^a^	−0.48^a^	−0.22^b^	0.05		0.012
** Δ** ***G*** **_t_ (%)**	−18.2^a^	−16.8^a^	−7.3^b^	1.4		0.002
**Short-circuit current (** ***I*** **_sc_)**						
** Basal ** ***I*** **_sc_ (μA/cm** 2 **)**	2.12	1.95	1.43	0.18		0.277
***E. coli*** ** 1 or ** ***E. coli*** ** 2 application** 3						
**Δ** ***I*** **_sc_ after 1 h (μA/cm** 2 **)**	−0.88^a^	−1.03^a^	−0.30^b^	0.16		0.074
** Δ** ***I*** **_sc_ (%)**	−31.9^a^	−51.3^a^	−6.7^b^	10.3		0.069

a,b Values within one row that do not share a common letter are different (P<0.05; Duncan's test).

1 G_t_ or I_sc_ at time zero is the basal value before addition *Escherichia coli.*

2 Data are arithmetic means and pooled standard error of means SEM [n =  30 (number of experiments for each treatment)].

3 Delta-values represent the absolute and relative changes of *G*_t_ and *I*_sc_ from 1 min before application to 60 min after application.

*E. coli* IMT11322 = avain non-pathogenic *E. coli*; *E. coli* IMT4529 = avain pathogenic *E. Coli*.

### Responses to Cholera Toxin, Histamine and Carbamoylcholine (Carbachol) Application

The responsiveness of the *E. coli*-infected intestinal tissues to cholera toxin, histamine and carbachol was investigated in a second step. Data regarding the effect of *E. coli* exposure on cholera toxin-induced electrophysiological variables are presented in Figure 1. The *G*_t_ of tissues pre-incubated with *E. coli* did not respond to the cholera toxin. However, the *G*_t_ of the control tissues increased after exposure to the cholera toxin. Furthermore, the addition of cholera toxin to the luminal side of isolated jejunal mucosa after pre-exposure to pathogenic *E. coli* caused an increase in *I*_sc_ but did not reach to the significance, while there was no such increase upon addition of cholera toxin after pre-exposure to non-pathogenic *E. coli* or control tissues. These results indicated that the pathogenic *E. coli* significantly increased the responsiveness to the cAMP/PKA-dependent secretagogue cholera toxin.

Serosal histamine application increased the *G*_t_ only after pathogenic *E. coli* exposure while no such increase was observed in uninfected control tissues or after exposure to non-pathogenic *E coli* (Figure 2). However, serosal histamine application did not elicit an increase of *I*_sc_ in any of the treatment, including pre-exposure to pathogenic *E. coli.*

Similarly to histamine, serosal carbachol application increased the *G*_t_ only after pre-exposure to pathogenic *E. coli* (*P*<0.05), while such increase was not present or not significant after infection with non-pathogenic *E coli* and in uninfected control tissues (Figure 3). A significant sensitivity of *I*_sc_ to carbachol was only evident in uninfected control segments (*P*<0.05). Both pathogenic and non-pathogenic *E. coli* blunted the *I*_sc_ responses to carbachol to non-significant levels, indicating that *E. coli* diminished the responsiveness to the Ca^2+^/PKC-dependent secretagogue carbachol.

## Discussion

The intestine is the most important reservoir of avian pathogenic *E. coli* [14]. In this context knowledge to understand how this pathogen affects the intestinal tract during colonization of the host is of special importance. Therefore, the present study was designed to investigate how the exposure to pathogenic and non-pathogenic *E. coli* might affect the active transepithelial ion transport and ionic permeability of the chicken gut.

In the present study, both *E. coli* strains induced decreases in the transmural conductance (*G*_t_) and *I*_sc_ in the jejunum of broiler chickens in comparison to uninfected control tissues. The decrease in *G*_t_ indicated that the jejunal epithelia became tighter to passive ion permeation after exposure to *E. coli*. A parallel change of *I*_sc_ indicates that the closure of cellular ion channels might have contributed to the decrease in *G*_t_; however, the magnitude of the Δ*I*_sc_ after *E. coli* infection was extremely small (∼1 μA), suggesting that the involvement of the cellular ion channels in the observed *G*_t_ response was only minor at best. Therefore the decrease in *G*_t_ should primarily reflect a decreased paracellular conductance, i.e., a tighter paracellular barrier.

In contrast to our results in chicken, it was shown in mammals that entero-pathogenic *E. coli* increased the tissue permeability [32], [33]. Roxas et al. [34] reported changes in intestinal ion permeability in the colon of mice infected with enterohemorrhagic *E. coli*, which correlated with alterations in tight junction architecture. Immunofluorescence microscopy revealed a redistribution of the tight junction proteins occludin and claudin-3 and increased expression of claudin-2 upon *E. coli* infection. Our observations indicate the differences in pathogenicity of *E. coli* strains in chickens might originate from differences in the host responses. It was hypothesized that this could be related to the innate intestinal immunity as the immune response to infection can affect other physiological functions [35], [36]. Basic physiological responses of epithelial cells, including changes in ion transport and subsequent fluid secretion, can play a crucial role in functional immunity and are utilized for the elimination of the invading pathogen [37]. Our results suggest that a key feature of functional epithelial immunity in chicken may be a tightening of the epithelium upon pathogen exposure. A similar tightening was seen previously for another Gram-negative bacterium, *Salmonella*, and could be attributed to epithelial recognition of its endotoxin [26]. Furthermore, Hering et al. [38] found that the TcpC protein of *E. coli* Nissle decreased the tissue conductance and affected the intestinal barrier function through upregulation of the tight junction protein claudin-14 in human epithelial HT-29/B6 cell culture, indicating the role of the innate immunity in the intestinal response. Thus, the different mucosal response to bacterial stimuli results from a different immune response towards intestinal pathogens.

The TLR signaling pathway plays a vital role in resistance to infection [39] and differences in this pathway between chicken and mammals could be involved in the different *G*_t_ responses of avian and mammalian intestine towards Gram-negative infections. Lotz et al. [40] found that TLR4 is the classical way of endotoxin signaling and leads to an increase in the tissue conductance in mammalian intestinal epithelial cells upon endotoxin stimulation. However, TLR2 has the opposite effect; it enhances the epithelial barrier and decreases the tissue conductance [41], [42]. Therefore, the response of the chicken intestine to *E. coli* exposure with a decrease in passive ion permeation may point to an enhancement of TLR2 relative to TLR4 signaling in this species [26].

A dominant way of intestinal pathogen elimination in mammals is diarrhea. One possible mechanism to induce diarrhea is transepithelial fluid secretion in response to certain inflammatory mediators (e.g., histamine) [43], neurotransmitters (e.g., acetylcholine, histamine) [44], or enterotoxins of the infecting bacteria (e.g., cholera toxin) [45]. The osmotic drag of water in such “secretory-type” diarrhea is caused by chloride secretion into the intestinal lumen which can be evidenced in Ussing chambers by an increased *I*_sc_ [46], [47]. In general, such increases in *I*_sc_ were either not present or very small after application of any of the three secretagogues used in the present study. This may suggest that chloride-driven secretory-type diarrhea is not a major mechanism to clear *E. coli* or Gram-negative bacteria in general from the chicken jejunum. Supportive evidence for this postulate comes from studies by Grubb et al. [48] who reported comparably low chloride transport rates with no significant net Cl^−^ transport across any region of the intestine of healthy chickens. Furthermore, in our recent study, it was shown that the Cl secretory function increased in the damaged mucosa of chickens due to a higher expression of the chloride channel ClC-2 [49]. Caldwell et al. [50] suggested that the epithelial ion secretion is a potential mechanism of immunity associated with host responses in the intestinal immune system of poultry, which may be important for the persistent colonization of pathogens in the gastrointestinal tract. Thus, the different outcomes of cholera toxin or *E. coli* enterotoxin in chickens versus humans is governed by host factors. Furthermore, the chloride ions can be transported across the intestinal epithelium by different pathways. It was shown that apical K^+^ channels could mask a secretory response and it may only be visible after blocking of apical K^+^ channels [51]. Therefore, further investigations are required to elucidate the mode of action.

Nonetheless, fluid accumulation has been observed in isolated jejunal loops of chicken after prolonged (18 h) exposure to cholera toxin [52]. Since the present study could not demonstrate a marked secretory response to cholera toxin, this fluid accumulation *in vivo* could alternatively be attributable to vascular damage induced by cholera toxin, leading to hyperfiltration of plasma water into the intestinal lumen (i.e., filtration-type diarrhea) [53]. Fluid filtration may be supported by an opening of intestinal epithelial tight junctions in response to cholera toxins as suggested by the increase in *G*_t_ after cholera toxin application in uninfected tissues. However, such increase in *G*_t_ was not evident after pre-incubation with *E. coli*, suggesting that the tightened epithelial barrier after *E. coli* infection includes protection against the barrier opening properties of cholera toxin. Chickens might prevent the toxic effects of the cholera toxin and enterotoxin through competition at the receptor level by covering tissue receptors such as ganglioside M1 (GM1) with some agent to antagonize toxin activity [54]. In addition, it was reported that the binding of the cholera toxin or *E. coli* enterotoxin to GM1 receptors alone is not sufficient to trigger the signaling events responsible for the intestinal hypersecretory processes [55]. In contrast to cholera toxin, histamine and carbachol selectively increased *G*_t_ but only in the co-presence of pathogenic *E. coli*. A similar dependence of the *G*_t_ response to histamine on the co-presence of pathogenic bacteria has been described earlier by our group with regard to *Salmonella* Enteritidis [26]. This may indicate that endogenous inflammatory and neuronal mediators may partly re-open a tightened epithelial barrier during pathogen infection.

While histamine did not induce any secretory response in the present study, carbamoylcholine was effective to increase *I*_sc_ slightly in uninfected control tissues. Comparable small responses to carbachol were also observed in previous studies on chicken jejunum [56], [57], indicating that such small secretory responses are typical for this segment of the chicken gut. Infection with *E. coli* reduced this response even further, indicating that both pathogenic and non-pathogenic *E. coli* interfere with carbachol signaling. This suggestion is in agreement with findings of Sanni et al. [31] that a haemolytic strain of *E. coli* infection also reduced the contractile responses to carbachol and histamine in duodenal smooth muscles of infected broilers.

Furthermore, the jejuna mucosa of chicken appears to be less sensitive to the secretory (*I*_sc_) effects of classical secretagogues. Nonetheless, effects on *G*_t_ demonstrate that receptors for these secretagogues and signaling cascades are either present or inducible by enteric infections. Taken together, the results of the present study indicated that both pathogenic and non-pathogenic *E. coli* strains reduced the ion permeability of jejunal epithelia immediately after exposure, suggesting that a decrease in ion permeability is a general response to *E. coli*, rather than a specific pathogen-induced response. Furthermore, the fast decrease in *G*_t_ suggests that the contact of the epithelium with *E. coli* or its endotoxin might be sufficient to elicit this effect.

Based on the results of the present study and our previous study [26], this response may represent a common reaction during Gram-negative infections or endotoxin release which might be directed towards a tightening of the epithelial barrier.

Future researches with the aim to discover the molecular basis of the differential responses of the chicken intestine to pathogenic infections are needed. In this regard, one key question is whether different parts of the chicken intestine respond differently to infection. A more detailed knowledge on how the avian host responds to intestinal infections can form the starting point for targeted gene studies.
